# Development and validation of classifiers and variable subsets for predicting nursing home admission

**DOI:** 10.1186/s12911-017-0442-4

**Published:** 2017-04-13

**Authors:** Mikko Nuutinen, Riikka-Leena Leskelä, Ella Suojalehto, Anniina Tirronen, Vesa Komssi

**Affiliations:** 1 Nordic Healthcare Group, Vattuniemenranta 2, Helsinki, 00210 Finland; 2 City of Tampere, PL 487, Tampere, 33101 Finland

**Keywords:** Nursing home admission, Classifier, Classification accuracy, Variable selection

## Abstract

**Background:**

In previous years a substantial number of studies have identified statistically important predictors of nursing home admission (NHA). However, as far as we know, the analyses have been done at the population-level. No prior research has analysed the prediction accuracy of a NHA model for individuals.

**Methods:**

This study is an analysis of 3056 longer-term home care customers in the city of Tampere, Finland. Data were collected from the records of social and health service usage and RAI-HC (Resident Assessment Instrument - Home Care) assessment system during January 2011 and September 2015. The aim was to find out the most efficient variable subsets to predict NHA for individuals and validate the accuracy. The variable subsets of predicting NHA were searched by sequential forward selection (SFS) method, a variable ranking metric and the classifiers of logistic regression (LR), support vector machine (SVM) and Gaussian naive Bayes (GNB). The validation of the results was guaranteed using randomly balanced data sets and cross-validation. The primary performance metrics for the classifiers were the prediction accuracy and AUC (average area under the curve).

**Results:**

The LR and GNB classifiers achieved 78% accuracy for predicting NHA. The most important variables were RAI MAPLE (Method for Assigning Priority Levels), functional impairment (RAI IADL, Activities of Daily Living), cognitive impairment (RAI CPS, Cognitive Performance Scale), memory disorders (diagnoses G30-G32 and F00-F03) and the use of community-based health-service and prior hospital use (emergency visits and periods of care).

**Conclusion:**

The accuracy of the classifier for individuals was high enough to convince the officials of the city of Tampere to integrate the predictive model based on the findings of this study as a part of home care information system. Further work need to be done to evaluate variables that are modifiable and responsive to interventions.

## Background

It is a common goal for elderly care services to support and enable living at home as long as possible. Most people would rather live at home in a familiar environment, than move to a nursing home or an assisted living facility. Also, from the view point of the service system, 24 h services are expensive. Thus, supporting functional and cognitive capabilities, which enable living at home, improves the quality of life and is cost saving for the payer. However, interventions aimed at improving or sustaining functional and cognitive capabilities can be expensive. Therefore, the need to be targeted to those individuals, who are at risk of needing 24 h service in the near future, but who are still capable of benefiting from the intervention. Furthermore, the resource planning of 24 h services benefits from the information of upcoming admissions. Identification of reliable predictors and creation of tools that calculate risk for individuals provide an answer to these problems.

Much research has focused on identifying predictors of nursing home admission (NHA) [1–14]. The studies vary according to variables, populations (e.g. with dementia [4, 8], without dementia [5, 11]), geographical locations (e.g. German [1], Singapore [12], Norway [15]) and sample sizes (e.g. *n* = 210 [1] or *n* = 7000 [9]) used in the determination of predictors of NHA. The commonly recognized risk factors include advanced age, functional and cognitive impairments, depression, caregiver burden, use of health services, prior hospitalization or nursing home use and dementia. In a literature review [5] and meta-analysis [16], the strongest predictors of NHA were increased age, low self-rated health status, functional and cognitive impairment, dementia and prior NHA.

The above research has focused on finding risk factors for NHA. However, as far as we know, no prior research has proved the prediction performance or accuracy of a NHA model for individuals. The prior research articulates the statistically important variables that increase or decrease the risk of NHA at the population level. It is based on the traditional statistical data processing approach in which statistical modeling connects data to a population of interest. It does not answer the question of how accurately the nursing home admission is possible to predict for individuals.

In this study, we point out the most important variable subsets of different sizes for predicting NHA. Particularly, we measure and validate the performance of our NHA prediction models in terms of classification accuracy. That is, we search the best model and measure how good it is for individuals. The variable subsets were searched by machine learning (ML) methods and the classification accuracy was calculated using the cross-validation principle. The data was consisted of the service records of home care clients in the city of Tampere^1^, Finland. Because our data set is highly unbalanced, we use a random operator to form balanced data sets and the all performance results are reported on those balanced data sets instead of the original unbalanced set.

We claim, that the knowledge of classification accuracy is highly valuable, when deciding on the adoption of the prediction model in actual service production. It should be noted, that statistically significant variables do not guarantee high classification accuracy. Without adequate accuracy, the cost effectiveness of the targeted interventions is not good enough: interventions are targeted to a significant number of people not at risk (“false positive”), and some of those in need of an intervention do not receive one (“false negatives”). Furthermore, the resource planning of service production benefits from the individual predictions of upcoming admissions. The primary contributions of this paper are summarized below:

- As far as we know, no prior research has investigated variable subsets of different sizes for predicting NHA. A few scholars have applied variable selection methods [3–5, 13]. However, they did not investigate the variable subsets of different sizes (1−*n* variables), as we did in this study.
- The second contribution relates to the way to use, train and validate classification algorithms for predicting NHA. Compared to prior research work, the present study investigates the NHA prediction models for individuals. Prior research investigated statistical significant population-level risk factors for NHA. The 5% level of significance was a de facto standard for important variables. In this study, we measure classification accuracy for classifiers trained and validated using cross-validation. That is, we study the accuracy of our model for unseen clients of home care according to the risk of NHA.

The objective of this study was to gain a better understanding of the accuracy level in which NHA can be predicted in order to support decision making in home care services and allocation of resources between customers. The classification accuracy of our method was 78% that was high enough for the decision to integrate it in the local information system of home caring^2^. The remainder of this paper is divided into three parts. In the first part, we describe how the variables of our prediction models were aggregated and how the variables were selected for the subset selection process. The second part introduces the methods for training and validating classifier algorithms. The third part of the paper presents the performance of the variable selection and discusses the results and practical implications.

## Methods

### Data source

The data consisted of the records of 7259 home care customers between January 2011 and September 2015 in the city of Tampere, Finland. These data were linked to records that contained information regarding all social and health care service usage during the same period. Nursing home admission (model outcome) was indicated by whether the customer admitted to a nursing home or not, and coded as a binary indicator. The data were linked on the customer level using unique encrypted identifiers. We excluded clients with recorded home care episode shorter than 12 months between January 2011 and September 2015 (*n*=3192) and those whose RAI-HC (Resident Assessment Instrument - Home Care [17]) values (*n*=981) were missing. In total, we had 3056 customers (539 NHA is “true” and 2544 NHA is “false”) for analysis.

**Fig. 1:**
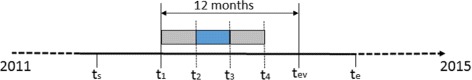
Variables were calculated in time periods of *t*_1_ - *t*_4_, when *t*_*s*_ - *t*_*e*_ is the time period of home care for a customer. Figure shows time scale of starting day and ending day of home care for customer i from which the variables of the models were derived

All the variables were calculated 3–12 months before the evaluation day *t*_*ev*_. In addition, the variables were calculated 6–12 months before the day *t*_*ev*_ for additional analyses. The main variables are listed in the column “variable” of Table 1. The variables were selected by the experts of elderly care services. Figure 1 shows a time scale in which *t*_*s*,*i*_ is the starting day and *t*_*e*,*i*_ the ending day of home care according to the home care service data for customer *i* (*i*=1,…,3056). The variables were the numbers of events or boolean value [*t* *r* *u* *e*/*f* *a* *l* *s* *e*] that an event occurred between times *t*_*k*_ and *t*_*k*+1_ (*k*=1,2,3) or *t*_1_ and *t*_4_. For example, variable *j* (a blue box in Fig. 1) for customer *i* was calculated from time period *t*_2,*ij*_−*t*_3,*ij*_. The interval between times *t*_1_ and *t*_*ev*_ was set to be 12 months and *t*_1_<*t*_2_<*t*_3_<*t*_4_<*t*_*ev*_. If NHA variable was “true” for the customer *i*, that is the customer *i* was admitted to nursing home at time *t*_*e*,*i*_, time *t*_*ev*,*i*_ was set to be the admission day (→ *t*_*ev*,*i*_=*t*_*e*,*i*_). If the NHA variable of customer *i* was “false”, then time *t*_*ev*,*i*_ was a random day between times *t*_*s*,*i*_ and *t*_*e*,*i*_, st. *t*_*ev*,*i*_>*t*_*s*,*i*_+12 months.

**Table 1:** The characteristics of the study sample (means / %) with and without a nursing home admission and the results of t-tests of significance difference between the means of continuous values or categorical variables

Variable	Time interval / description	NH admission	No NH admission	*p*-value
Age (mean)		84.44	81.76	<.0001
Number of Emergency care visits (mean)	3-6 months	0.72	0.35	<.0001
	6-9 months	0.78	0.38	<.0001
	9-12 months	0.61	0.37	<.0001
Number of emergency care visits, change (mean)	3-6 months vs. 6-9 months	-0.06	-0.03	.6279
	6-9 months vs. 9-12 months	0.18	0.01	.0049
Number of periods of care (mean)	3-6 months	0.98	0.36	<.0001
	6-9 months	0.86	0.32	<.0001
	9-12 months	0.53	0.33	.0002
Number of periods of care, change (mean)	3-6 months vs. 6-9 months	0.12	0.03	.3121
	6-9 months vs. 9-12 months	0.33	-0.01	<.0001
Number of home care visits (mean)	3-6 months	131.51	96.30	<.0001
	6-9 months	142.80	93.42	<.0001
	9-12 months	130.10	89.26	<.0001
Number of home care visits, change (mean)	3-6 months vs. 6-9 months	-11.28	2.88	.0002
	6-9 months vs. 9-12 months	12.70	4.16	.0118
Number of outpatient visits in	3-6 months	1.89	1.11	<.0001
specialised care by appointment (mean)	6-9 months	1.77	1.14	<.0001
	9-12 months	1.58	1.16	.0001
Number of outpatient visits in specialised	3-6 months vs. 6-9 months	0.12	-0.03	.1410
care by appointment, change (mean)	6-9 months vs. 9-12 months	0.19	-0.02	.0588
Number of physiotherapy visits at home (mean)	3-12 months	0.47	0.49	.9006
Number of outpatient visits in geriatrics (mean)	3-12 months	1.56	.62	<.0001
Number of physician visits at home (mean)	3-12 months	0.57	0.49	.1316
RAI-HC (mean)^a^	CPS	1.50	0.92	<.0001
	IADL	13.18	9.34	<.0001
	PAIN	0.72	0.77	.2278
	MAPLE	4.06	3.14	<.0001
Customer of support service (%)	Safety phone	41%	25%	<.0001
	meals-on-wheels	47%	28%	<.0001
	shopping	38%	27%	<.0001
	Cleaning	10%	10%	.9058
	transportation	29%	15%	<.0001
	Day center	30%	15%	<.0001
	Support for informal care	5%	5%	.9691
	Home rehabilitation	4%	3%	.3683
Outpatient visit in specialised care (%)	Surgery / neurosurgery	29%	13%	<.0001
	Internal medicine	40%	22%	<.0001
	Obstetric	3%	2%	.0367
	Neurology	17%	9%	<.0001
	Respiratory medicine	4%	4%	.8502
	Ophthalmology	12%	7%	.0004
	Phoniatrics	14%	5%	<.0001
	Psychiatry	14%	9%	.0004
Period of care in specialised care (%)	Surgery / neurosurgery	20%	8%	<.0001
	Internal medicine	30%	16%	<.0001
	Obstetric	0%	0%	.5341
	Neurology	10%	3%	<.0001
	Respiratory medicine	2%	2%	.5684
	Ophthalmology	1%	0%	.0028
	Phoniatrics	1%	0%	.1042
	Psychiatry	8%	2%	<.0001
	Intensive care unit	1%	0%	.0211
Diagnosis (%)	a00-a09	4%	2%	<.0001
	a30-a49	4%	3%	.1679
	e00-e07	3%	2%	0.0723
	e10-e14	9%	6%	.0037
	e70-e90	5%	2%	<.0001
	f00-f03	50%	16%	<.0001
	f04-f09	4%	2%	.0006
	f10-f19	2%	1%	.3515
	f20-f29	2%	2%	.7376
	f30-f39	6%	4%	.0123
	g20-g26	5%	2%	.0001
	g30-g32	32%	6%	<.0001
	g40-g47	3%	2%	.0972
	i10-i15	28%	12%	<.0001
	i20-i25	14%	6%	<.0001
	i30-i52	25%	13%	<.0001
	i60-i69	9%	4%	<.0001
	i70-i79	3%	1%	.0023
	i80-i89	2%	1%	.1083
	i95-i99	5%	1%	<.0001
	j09-j18	6%	3%	.0136
	j20-j22	3%	2%	.0437
	j40-j47	5%	3%	.1991
	k55-k63	5%	2%	<.0001
	m05-m14	2%	2%	.7376
	m15-m19	5%	2%	.0005
	m45-m49	2%	1%	.0008
	m50-m54	4%	2%	.0933
	m70-m79	3%	2%	.0978
	m80-m85	4%	2%	.0008
	n10-n16	4%	2%	.0324
	n17-n19	5%	2%	.0001
	n30-n39	15%	5%	<.0001
	n40-n51	2%	1%	.0813
	r00-r09	2%	2%	.9037
	r10-r19	4%	2%	.0034
	r40-r46	5%	2%	.0012
	r50-r69	10%	4%	<.0001
	s00-s09	8%	2%	<.0001
	s30-s39	2%	1%	.0115
	s40-s49	2%	1%	.3970
	s70-s79	5%	2%	<.0001
	s80-s89	2%	1%	.1098
	z00-z13	5%	3%	.1123

^a^Resident Assessment Instrument for Home Care (RAI-HC). The Cognitive Performance Scale (CPS) uses items on memory and communication skills to create a 7-point scale from 0 (intact) to 6 (very seve re) [35]. The Instrumental Activities of Daily Living (IADL) scale [36] provides a measure of the customer’s self-performance of seven daily tasks: meal preparation, ordinary housework, managing finances, managing medications, phone use, shopping and transportation. The scores are from 0 to 21. The Method for Assigning Priority Levels (MAPLe) differentiates customers into five different groups ranging from low to very high risk of health decline [34]. Higher risk group indicates a higher risk to be admitted to a long-term care facility

Table 1 presents general characteristics of the study sample (*n*=3056), of which 539 (17.6%) were admitted to nursing home. The table includes the results of t-test of significance difference for continuous variables and chi-squared test for categorical variables between the groups of home caring customers and nursing home residents.

### Variable subset selection

The aim of this study was to find efficient variable subsets **X**_*sub*_={*x*_*i*_|*i*=1,…*n*} from a large variable set **X**={*x*_*j*_|*j*=1,…*k*} for predicting the NHA when *n* and *k* are the numbers of variables and *n*<*k*. Let *Y* be the binary vector of NHA variable, *F*(·) is a classifier and *Y*=*F*(**X**_*sub*_). That is, we predict the state of *Y*_*i*_ for customer *i* at time *t*_*ev*,*i*_ (Fig. 1), when the variable vector *X*_*sub*,*i*_ is calculated from time range *t*_1,*i*_−*t*_4,*i*_ (3–12 months before *t*_*ev*,*i*_) or *t*_1,*i*_−*t*_3,*i*_ (6–12 months before *t*_*ev*,*i*_).

Variable selection is a mature research topic and has been used for many applications [18]. In this study we applied sequential forward selection (SFS) method [19] for variable subset generation. SFS starts with an empty set and adds one variable at a time from the original set **X** for classifier by maximizing the performance measure. Our primary performance metric was classification accuracy:

1 $$ acc=\frac{1}{n_{samples}} \sum_{i=1}^{n_{samples}} L\left(y_{pred,i}=y_{i}\right)   $$

where *y*_*pred*,*i*_ is the predicted NHA class of the *i*-th sample, *y*_*i*_ is the corresponding true NHA class, *n*_*samples*_ is the number of samples and *L*(·) is the indicator function (*L*=1 if *y*_*pred*_=*y*; *L*=0 if *y*_*pred*_≠*y*) [20]. We additionally calculated the average area under the curve (AUC) and true-positive rate (recall) values for classifiers. AUC values correlated almost perfectly with *acc* values, but we decided to report them, because in some research areas they are more familiar than the *acc* values. Recall of a classifier is calculated by dividing the correctly classified positives (true positives) by the total positive count (true positives + false negatives) [21]. That is, recall is the probability that a risk customer is found.

The strength of the accuracy metric, compared to the other common metrics, is that the accuracy metric is easy to understand. It should be noted, that our data set is highly unbalanced. We use a random operator to form balanced data sets and the performance results are reported on those balanced data sets instead of the original set. Otherwise, the accuracy metric would be biased and not suitable.

An alternative of SFS would be sequential backward elimination (SBE). SBE starts with **X** and eliminates one variable at a time by maximizing the performance measure. Our selection of SFS instead of the SBE method is justified by the ratio of relevant (*#* *r*) and all (*k*) variables. According to Liu et al. [18], if *#* *r* is small, then the SFS strategy should be used, and if the number of irrelevant variables (*k*−*#* *r*) is small, then the SBE strategy should be used. According to the pre-tests, the original variable set **X** includes many irrelevant variables (low univariate prediction power); thus, we prefer the SFS strategy.

**Fig. 2:**
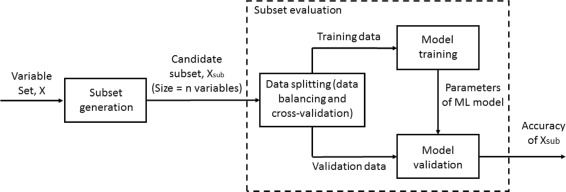
The framework of variable subset selection used in this study. The subset generation component feeds candidate variable subset to subset evaluation component. Evaluation component trains and validates classifier and calculates the accuracy values for the subset

Different classifiers have different performance for different data sets. In this study, we evaluated the performance of three classifiers: logistic regression (LR) [22], Gaussian naive Bayes (GNB) [23] and support vector classifier (SVC) [24]. That is, SFS was run three times using the classifiers of LR, GNB or SVC. Figure 2 shows the components of variable subset selection process. The subset generation component (SFS) feeds candidate variable subset **X**_*sub*_ to subset evaluation component. Evaluation component trains and validates classifier and calculates the accuracy values for the subset **X**_*sub*_.

It should be noted, that we use a random operator to form balanced data sets for the analyses. Let *A*={**X**|*Y*=1} and *B*={**X**|*Y*=0} be the data sets. That is, the set *A* contains the data of customers with the values of “true” of NHA variable and the *B* with “false”. Because the set *A* is smaller (*n*=539) than the set *B* (*n*=2517), the balanced data set *C* was formed, st. *C*=*A*∪*R*(*B*) where *R* is a random operator for selecting 539 random samples from *B*, thus setting the level of chance at 50%. To be sure that the selection did not bias the results, data set *C* was formed 100 times.

Furthermore, classifier algorithms were trained and validated using a ten-fold cross validation method. That is, we formed sample *C*_*i*_ (*i*=1,…,100) from the data sets *A* and *B*, and split it into 10 equal-sized parts *P*_*ik*_ (*P*_*ik*_∈*C*_*i*_ and *k*=1,…10). The classification accuracy value, *a* *c* *c*_*ik*_, was calculated by Eq. 1 for the part *P*_*ik*_ of the data set *C*_*i*_ when the parameters of the classifier were trained with the other K-1 parts of the data set *C*_*i*_. The process was repeated for *k*=1,2,…10. The overall classification accuracy, *CA*, for the subset **X**_*sub*,*n*_ of size n (*n*=1,…15) was calculated as

2 $$ CA_{\textbf{X}_{sub,n}}=\frac{1}{100*K} \sum_{i=1}^{100} \sum_{K=1}^{10} acc_{ik}   $$

SFS calculated the best variable subsets for all balanced data sets *C*_*i*_. That is, we have 100 variable subsets of size of 1–15 variables. The (average) importance of each variable was measured by a rank metric:

3 $$ R(j)=\frac{1}{100} \sum_{i=1}^{100} \#F-r(i,j)   $$

where *r*(*i*,*j*) is the rank of variable *j* based on sample *C*_*i*_ and *#* *F* is the size of the largest subset that was formed by SFS [25–27]. In this study *#* *F*=15. Higher *R*(*j*) indicates that variable *j* is more important according to SFS, because it was selected for smaller size variable subsets. That is, variable has higher prediction capability according to SFS and its NHA classification ability is high.

### Software

We used four Python packages: *sklearn* [20], *mlxtend* [28], *numpy* [29] and *pandas* [30] to implement the classifiers and compute, *acc*, *AUC*, *recall*, *C* *A*_*Xsub*_ and *R*(*j*). SFS was computed by the function “SequentialFeatureSelector” in the package *mlxtend*. The classifiers of LR, SVC and GBN were implemented using the functions from the *sklearn.linear_model*, *sklearn.svm* and *sklearn.naive_bayes* packages. The packages of *numpy* and *pandas* were used for data reading and processing.

## Results

**Fig. 3:**
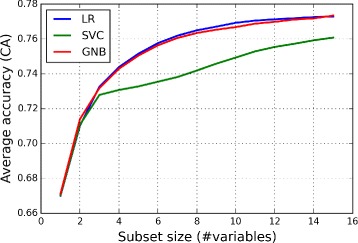
Average accuracy as a function of the size of variable subset. Figure shows the classification accuracy of the feature subsets found by different classifiers as a function of the subset size. The average classification accuracy values of LR and GNB methods differ from the SVC method

Figure 3 shows the performance (average classification accuracy) of the feature subsets found by different classifiers as a function of the subset size when the variables were calculated 3–12 months before the evaluation day *t*_*ev*_. The average classification accuracy values were determined by Eq. 2. The classifiers of LR, SVC and GNB had the average accuracies of 0.776 (*C* *I*95*%*=.0025), 0.762 (*C* *I*95*%*=.0026) and 0.776 (*C* *I*95*%*=.0024), respectively, for the variable subset of 15 variables. According to the student’s t-tests [31], the average classification accuracy value of LR and GNB methods with 15 variables differed statistically from the SVC method: LR vs. SVC (*p*<.0001) and GNB vs. SVC (*p*<.0001).

In addition, we calculated average AUC and recall values for the classifiers. The AUC values were 0.846 (*C* *I*95*%*=.0025), 0.838 (*C* *I*95*%*=.0025) and 0.847 (*C* *I*95*%*=.0024) for the classifiers of LR, SVC and GNB with 15 variables. The recall values were 0.755 (*C* *I*95*%*=.0015), 0.724 (*C* *I*95*%*=.0018) and 0.756 (*C* *I*95*%*=.0018). An AUC of 0.5 indicates no discrimination above chance and an AUC of 1.0 indicates perfect classification. A rough guide for the classification ability is AUC 0.9−1.0 excellent, AUC 0.8−0.9 good, AUC 0.7−0.8 fair and AUC 0.6−0.7 poor [32]. In general, classification ability is useful if AUC >0.75 [33]. That is, the performance of the classifiers with 15 variables was at good level.

When the variables were calculated 6–12 months before the evaluation day *t*_*ev*_, the average accuracy of classifiers of LR, SVC and GNB were 0.747 (*C* *I*95*%*=.0030), 0.737 (*C* *I*95*%*=.0029) and 0.734 (*C* *I*95*%*=.0029), respectively, for the variable subset of 15 variables. The AUC values were 0.819 (*C* *I*95*%*=.0027), 0.810 (*C* *I*95*%*=.0028) and 0.813 (*C* *I*95*%*=.0025). The recall values were 0.732 (*C* *I*95*%*=.0017), 0.738 (*C* *I*95*%*=.0025) and 0.732 (*C* *I*95*%*=.0026). The results of the 6–12 months variables show a moderate decrease in performance compared to the 3–12 months variables (e.g. LR CA: 0.776 → 0.747). The performance of the classifiers with the 6–12 months variables, however, is still at good level (*A* *U* *C*>0.8).

**Fig. 4:**
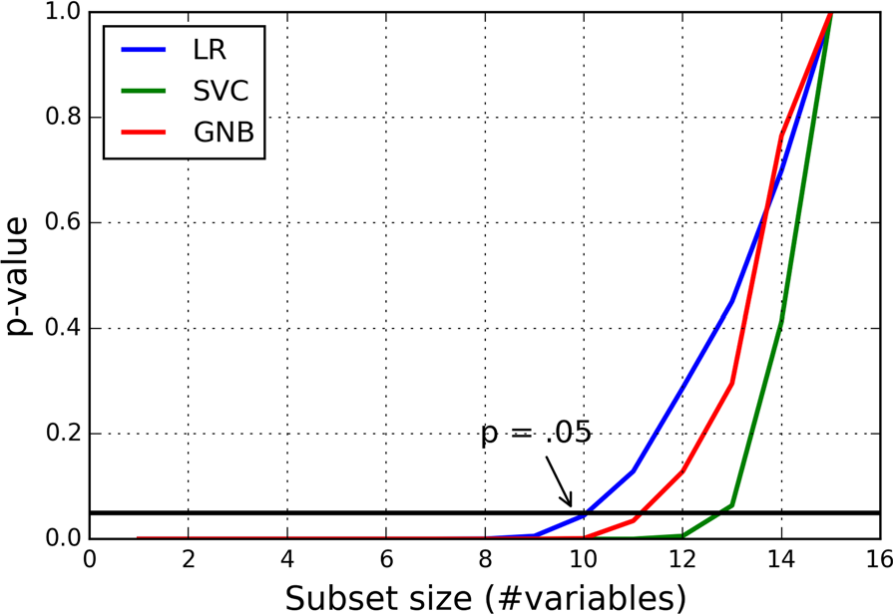
*P*-value as a function of the size of variable subset compared to the subsets of 15 variables. We defined that if *p*<.05, the difference between the performances of variable subsets is statistically significant. According to the definition, the optimal subset size for LR method is 9 variables. That is, the performance achieved by the subset size of 9 variables did not differ statistically from the subset of 15 variables, when the classifier is LR

Figure 4 shows the *p*-values calculated by the student’s t-test when the average classification accuracy values for the subsets of 15 variables of 3–12 months were compared to the subsets of *n* variables (*n*=1,…15). We defined that if *p*<.05, the difference between the performances of variable subsets is statistically significant. According to the definition, the optimal subset size for LR method was 9 variables. That is, the performance achieved by the subset size of 9 variables did not differ statistically from the subset of 15 variables, when the classifier was LR.

Table 2 sorts the variables according to ranking score, *R*, described by Eq. 3, for the classifiers of LR and GNB. Large *R*(*j*) value means that the variable *j* was selected regularly in small variable subsets for different balanced data sets *C*. That is, the NHA classification ability of the variable *j* is high. According to the results, the most important variables were the diagnoses of G30-G32 and F00-F03 and the RAI metrics of IADL (Activities of Daily Living), MAPLE (Method for Assigning Priority Levels) and CPS (Cognitive Performance Scale). In addition, variables related to the numbes of periods of care were important variables for predicting NHA with the both classifiers. It should be noted, that the RAI variables (IADL, MAPLE and CPS) are not simple measurements or observations, but instead scoring systems developed by researcher and practitioners (e.g., MAPLE [34], CPS [35] and IADL [36]). That is, it is not surprising that these variables have such high performance at predicting NHA.

**Table 2:** The 10 variables of the highest ranking score values calculated for the LR and GNB classifiers (the important variables for the both classifiers are marked as stars)

#	LR classifier: Variables	Ranking score
1	**Diagnosis F00-F03	147.9
2	**Diagnosis G30-G32	105.4
3	Number of periods of care (6-9 months)	99.9
4	**RAI IADL	85.5
5	**RAI CPS	73.9
6	**RAI MAPLE5	53.9
7	Number of Emergency care visits (3-6 months)	37.4
8	Diagnosis N30-N39	33.8
9	Diagnosis M15-M19	26.6
10	Number of periods of care (3-6 months)	26.1
#	GNB classifier: Variables	Ranking score
1	**Diagnosis F00-F03	147.2
2	**RAI IADL	106.9
3	**Diagnosis G30-G32	84.2
4	Number of home care visits, change (3-6 months vs. 6-9 months)	84
5	Number of periods of care, change (3-6 months vs. 6-9 months)	78.7
6	**RAI CPS	77.9
7	**RAI MAPLE5	68.8
8	RAI PAIN	57.9
9	Specialised care by appointment (6-9 months)	57
10	Specialised care by appointment (3-6 months)	44.9

**Fig. 5:**
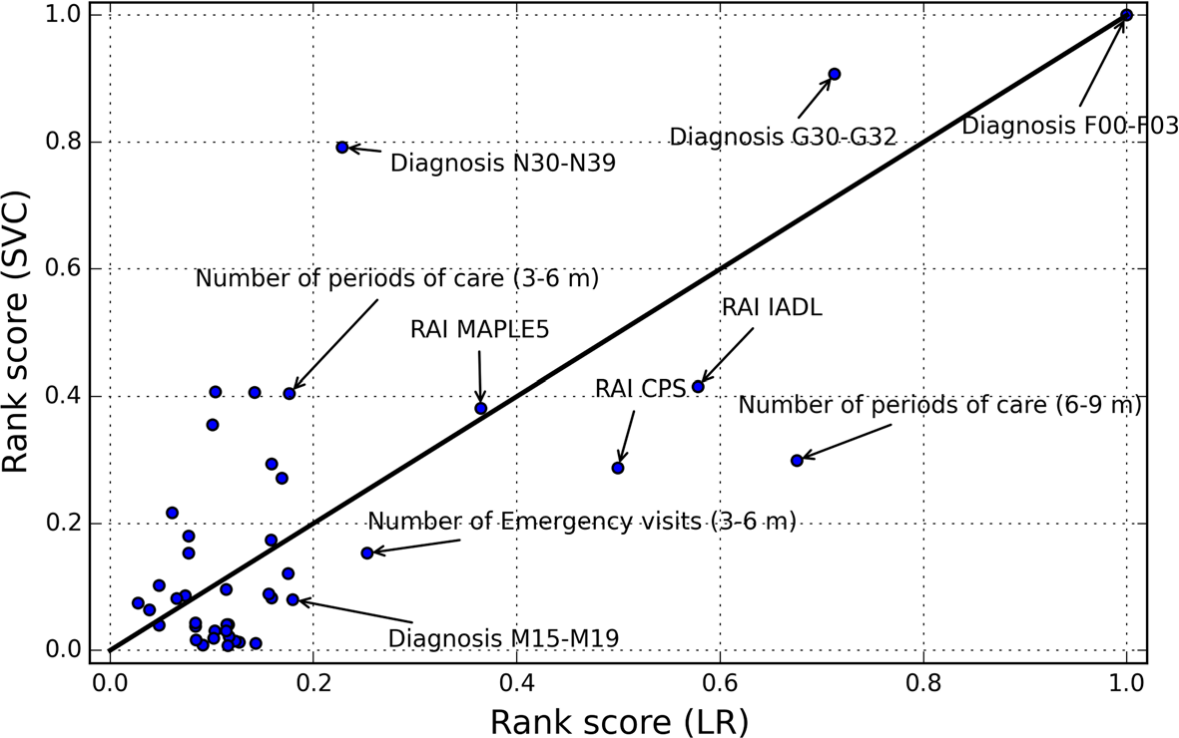
Normalized ranking score values of the SVC method as a function of the LR method. The variables of the RAI MAPLE, RAI IADL, RAI CPS and diagnoses F00-F03 and G30-G32 were five important variables for the both classifiers

**Fig. 6:**
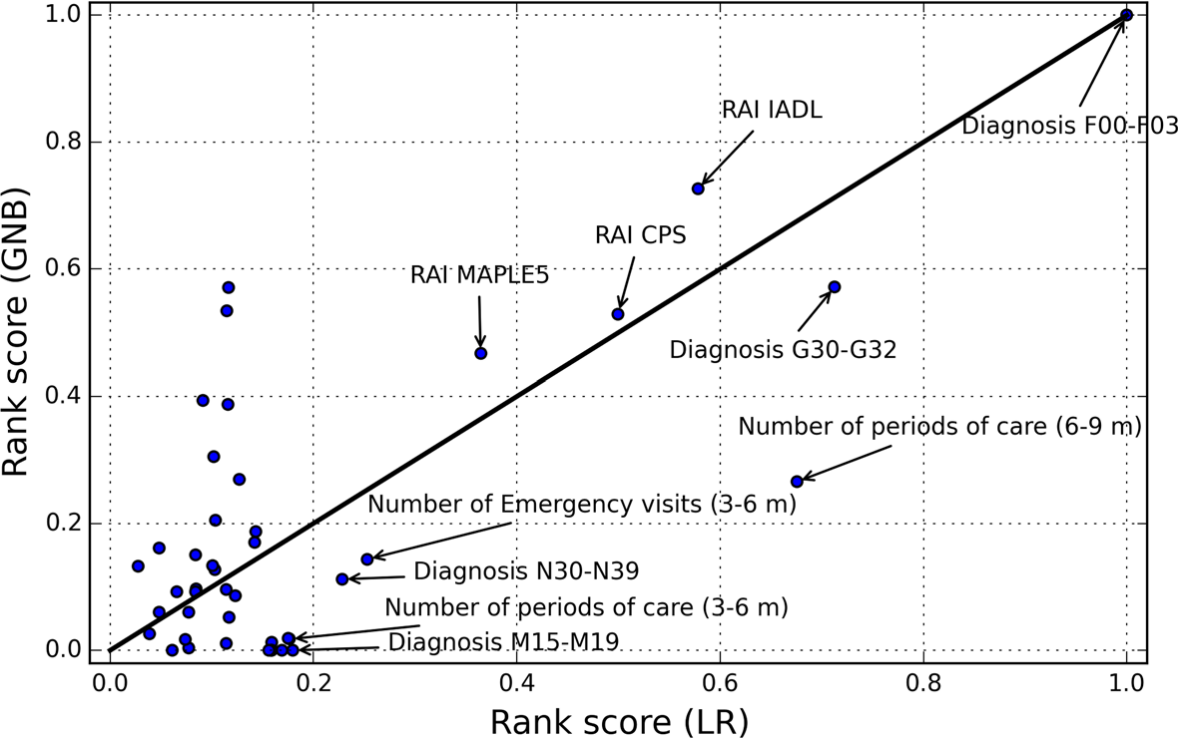
Normalized ranking score values of the GNB method as a function of the LR method. The variables of the RAI MAPLE, RAI IADL, RAI CPS and diagnoses F00-F03 and G30-G32 were five important variables for the both classifiers

Figures 5 and 6 plot the normalized ranking score values for the classifiers of SVC and GNB as a function of the values of LR. Ten variables with the highest *R* values of LR classifier are labelled on the figures. The 45° identity line visualizes the differences between the R values of the classifiers. Variables in the lower-right region of the line were more important for the LR than for the SVC (Fig. 5) or for the GNB (Fig. 6). Similarly, those in the upper-left region were more important for the SVC (Fig. 5) or for the GNB (Fig. 6) than for the LR. For example, the diagnosis N30-N39 was more important for the SVC classifier than for the LR. However, the differences between the most important variables of the classifier were rather small. The variables of the RAI MAPLE, RAI IADL, RAI CPS and diagnoses F00-F03 and G30-G32 were five important variables for the all classifiers.

## Discussion

The aim of the study was to analyse predictors and find out efficient variable subsets to predict NHA in a sample of home caring customers. Particularly, we wanted to find and report the level of accuracy in which NHA can be predicted for individuals. Our results show that the admission of nursing home can be predicted at an accuracy level of 78% / 74% when the variables were calculated 3–12 months / 6–12 months before the evaluation day. Thus, on average, our model predicts four out of five or three out of four home care customers in the right class in terms of nursing home admission. This is crucial information for decision makers for two reasons. Firstly, the model has to be accurate enough so that investments in preventive interventions can be made. If the accuracy of the model is too low, there are too many false positives and the cost effectiveness of the interventions is low. Secondly, the model needs to predict the individuals with high risk well in advance of the admission. Otherwise, it is too late to implement any interventions. Therefore, the fact that the accuracy of our model with variables 6–12 months before the evaluation day is as high as 74%, is important.

As far as we know, no prior research has published the classification accuracy of the NHA model for individuals. It should be noted, that the classification accuracy is a very common metric in machine learning and other fields. However, prior research has done the analyses at the population level. The important variables have been detected using the 5% level of significance. That is, the values of the parameters of a model (e.g. linear regression (e.g. [12]), logistic regression (e.g. [9, 14]) or Cox model (e.g. [4, 5])) are estimated from whole data (without the split of train and test sets) and the significance levels for coefficients are derived. Nothing else has done to see if the model generalizes on the data and individuals that played no role in estimating the parameters for models. Few scholars of NHA (e.g. [2, 12]) have applied goodness-of-fit tests (e.g. AIC, *R* ^2^) for the model, but the test results were often more close to zero than one (≈.20−.25).

We see that the above lacks in NHA research are related to the public health science and data modelling cultures, in which model validation is omitted or calculated only on training data [37]. In this study we searched the important variables by averaging the results of variable selection that was executed for many random split of the whole data set. The importance of variables was measured by the ranking metric. The level of classification accuracy of model for different variable subsets was tested by cross-validation. The variable selection from many random data samples and cross-validation warrants the generalization of our variables and models.

The variables of RAI MAPLE, functional impairment (RAI IADL), cognitive impairment (RAI CPS), memory disorders (G30-G32 and F00-F03) and the use of community-based health services and prior hospital use (emergency visits and periods of care) were the most important. The ICD10 (International Classification of Diseases) group of G30-G32 contains the codes for other degenerative diseases of the nervous system (e.g. Alzheimer) and F00-F03 for dementia. A comparison of our results with the findings of the other investigations revealed that especially, functional [1–3, 5–9, 11, 13, 14] and cognitive [2, 5, 8, 9, 11, 14] impairment, dementia [1, 3, 13, 14] and use of community-based health services [2, 4] or prior hospitalization [9] were also strong predictors of NHA. In contrast to our findings, [2, 4–6, 9, 13, 14] found that increased age lead to increased risk of NHA. In our study, the importance of variable of age was rather low according to the ranking score.

The major strengths of this study include its detailed assessment of important variables and model validation and availability of a range of important variables for nursing home admission. The accuracy of the model was high enough to convince the officials of the city of Tampere to integrate the predictive model as a part of home care information system. However, there are some limitations to the present study. We were unable to investigate the associations of social relationships with nursing home admission. Some studies have shown that caregiver characteristics [4, 7, 14, 38, 39], having children [8, 9] and marital status [6] can be important factors for NHA. Second, this was a study of home caring clients living in a defined geographical location, which may limit the generalizability to older adults living in other areas. Also the finding of this study may not be applicable to population without home caring services. In addition, many of the evaluated risk variables found in this study, are not modifiable. Further work need to be done to evaluate variables that are modifiable and responsive to interventions.

## Practical implications

It is clear, that applying ML methods will progress and reform the work of the gerontology researchers and practitioners. The benefits can be viewed from the two aspects: 1) ML methods can be used to construct practical computer software for predicting NHA to aid the decision-making of practitioners, 2) large variable groups can be studied and the most important variables can be found.

The aspect (1) contributes most to the work of practitioners, e.g. home care case managers. The problem the case managers face is equivalent to that in any preventive care: it is difficult to achieve cost-efficiency if you cannot target a specific subgroup. You usually provide a small intervention for everyone, which is not enough for those at high risk. In order to be effective, the preventive measure needs to be substantial (e.g. in the case of home care customers, 2000€) but becomes too expensive, if offered for many customers. With limited resources one needs to know which customers are most in need of a rehabilitation intervention and target those individuals to maximize cost-effectiveness.

In the case of home care in Tampere, about 17% of customers are admitted to a nursing home within a year, which is the a priori risk for everyone. The algorithm produced with ML techniques gives a much more accurate risk value enabling the targeting off interventions. Without an accurate prediction algorithm, it is difficult to identify the high risk individuals. It is not enough to identify variables that have a statistically significant relationship with NHA, because this does not provide guidelines that can be applied in practice. For example, we know that a diagnosis indicating dementia or Alzheimer’s disease increases NHA risk, but this information is not specific enough to identify the individuals in need of an intervention (unless we target everyone with that particular diagnosis). The ML algorithm provides a risk classification and also allows for the estimation of the accuracy of the prediction. Also, in many cases the case managers need to convince their superiors of the need of investing in rehabilitation interventions for a particular customer. The risk estimate from a validated prediction algorithm can be used as a means of communication between the case manager and her superior.

Furthermore, the prediction model can also be used to estimate resource requirements for 24 h services by summing up the individual predictions. The predictions provide an upper limit estimation for capacity requirement. With time, when data is gathered on by how much targeted rehabilitation interventions can reduce NHA, the capacity estimates become more accurate.

In this study, the city of Tampere integrated a computer software containing the prediction algorithm in their data warehouse. The computer software aggregates and processes the variables from different databases and calculates the customer specific NHA risk value. If the risk is high, the case managers consider customer specific interventions, e.g. a new service level assessment, more home care visits, a particular therapy or revised medication. Prior to the implementation of the prediction algorithm, the rehabilitation interventions were not targeted systematically. Most often interventions were used when a care taker or nurse or next of kin noticed a change in functional ability and notified the case manager. When using the prediction algorithm, interventions are targeted based on more objective evaluations and customers are screened regularly. This way it is possible to identify customers at risk earlier than before. Also, after the implementation of the prediction algorithm, the selection of different rehabilitation interventions available for home care customers has been increased.

The next step in the study and implementation project is to gather data from the interventions and their effects, and build another ML model to predict the effectiveness of each intervention for each type of customer. Also, the model can be used to predict, who is no longer capable of benefitting from an intervention. This added information will further improve the cost-effectiveness of home care.

The aspect (2) contributes both the gerontology research and practical work. Variable selection can be used to identify which of the available variables are closely related to the prediction of the NHA and to discard those unrelated to it, reducing the dimensionality of the dataset. For the researcher of gerontology, the process of variable selection may indicate new variables that had not been previously considered as relevant to NHA. For example, in this study, we found about 10 important variables for predicting NHA. Furthermore, the model validity is easier to evaluate after variable selection is used to reduce the dimensionality of the model. After dimension reduction, the researchers know the variables for which they should focus in their research [40]. For the NHA research, this may mean that the variables for which the interventions should be focused can be found.

The second benefit, because of the variable selection, is that the number of variables, integrated in the software tool, can be minimized. This is important, because each new added variable requires resources for the processes of data aggregation and validation and requirements for data integration from different databases.

## Conclusion

Most elderly people prefer to live at home in a familiar environment than move to a nursing home. The findings of our study indicate important variable subsets for predicting NHA of community dwelling home care customers, and offer potential to find those individuals at the level of 78%, who are at risk of NHA. The most important variables were RAI MAPLE, functional impairment (RAI IADL), cognitive impairment (RAI CPS), memory disorders (diagnoses G30-G32 and F00-F03) and the use of community-based health-service and prior hospital use.

## Endnotes

^1^ Tampere is the third largest city in Finland. The percent of population over 65 years is 18.0% that is approximately same as in the other big cities in Finland (http://www.stat.fi). Also, the scope or services offered for the elderly as well as eligibility criteria for home care and nursing home care are fairly similar in all areas in Finland.

^2^ Kotitori http://www.tampereenkotitori.fi/

## Abbreviations

AUC: Area under the curve
CA: Classification accuracy
CPS: Cognitive performance scale
GNB: Gaussian naive Bayes
IADL: Activities of daily living
LR: Logistic regression
MAPLE: Method for assigning priority levels
ML: Machine learning
NHS: Nursing home admission
RAI-HC: Resident assessment instrument - home care
SBE: Sequential backward elimination
SFS: Sequential forward selection
SVM: Support vector machine
